# Molecular and Clinicopathological Biomarkers in the Neoadjuvant Treatment of Patients with Advanced Resectable Melanoma

**DOI:** 10.3390/biomedicines12030669

**Published:** 2024-03-17

**Authors:** Piotr J. Błoński, Anna M. Czarnecka, Krzysztof Ostaszewski, Anna Szumera-Ciećkiewicz, Piotr Rutkowski

**Affiliations:** 1 Department of Soft Tissue/Bone Sarcoma and Melanoma, Maria Sklodowska-Curie National Research Institute of Oncology, 02-781 Warsaw, Polandkrzysztof.ostaszewski@nio.gov.pl (K.O.); or piotr.rutkowski@pib-nio.pl (P.R.); 2 Faculty of Medicine, Medical University of Warsaw, 02-091 Warsaw, Poland; 3 Department of Experimental Pharmacology, Mossakowski Medical Research Centre, Polish Academy of Sciences, 02-106 Warsaw, Poland; 4 Department of Pathology, Maria Sklodowska-Curie National Research Institute of Oncology, 02-781 Warsaw, Poland; anna.szumera-cieckiewicz@pib-nio.pl

**Keywords:** neoadjuvant immunotherapy, neoadjuvant-targeted therapy, predictive factors, prognostic factors

## Abstract

Neoadjuvant systemic therapy is emerging as the best medical practice in patients with resectable stage III melanoma. As different regimens are expected to become available in this approach, the improved optimization of treatment strategies is required. Personalization of care in each individual patient—by precisely determining the disease-related risk and the most efficient therapeutic approach—is expected to minimize disease recurrence, but also the incidence of treatment-related adverse events and the extent of surgical intervention. This can be achieved through validation and clinical application of predictive and prognostic biomarkers. For immune checkpoint inhibitors, there are no validated predictive biomarkers until now. Promising predictive molecular biomarkers for neoadjuvant immunotherapy are tumor mutational burden and the interferon-gamma pathway expression signature. Pathological response to neoadjuvant treatment is a biomarker of a favorable prognosis and surrogate endpoint for recurrence-free survival in clinical trials. Despite the reliability of these biomarkers, risk stratification and response prediction in the neoadjuvant setting are still unsatisfactory and represent a critical knowledge gap, limiting the development of optimized personalized strategies in everyday practice.

## 1. Introduction

The treatment of stage III resectable melanoma has evolved, as novel adjuvant systemic therapy options have been introduced [1]. Targeted therapy (TT) and immune checkpoint inhibitors (ICIs), which initially had been found to improve patient outcomes in a metastatic setting, were also shown to have significant efficacy in adjuvant treatment [2,3,4]. One year of adjuvant treatment with pembrolizumab (anti-PD-1) resulted in 59.8% recurrence-free survival at 3.5 years, compared to 41.4% in patients who received a placebo and 55.4% to 38.3% after 5 years, respectively [3,5]. Similarly, the adjuvant-targeted therapy with dabrafenib plus trametinib (BRAF and MEK inhibitors) in patients with *BRAF-*mutated tumors resulted in 58% relapse-free survival at 3 years, compared to 39% in the placebo group and 52% to 36% at 5 years, respectively [3,6]. Adjuvant systemic therapy with ICIs or targeted therapy has become the standard of care in patients with resectable stage III/IV melanoma [7,8]. More recently, in patients with high-risk resectable stage III/IV melanoma, neoadjuvant systemic therapy has emerged as even more beneficial than adjuvant treatment [9,10,11,12]. The advantages of the neoadjuvant over the adjuvant strategy include a reduction in tumor burden (thus, also extent of surgery), assessment of the pathological response, which is a reliable prognostic factor, and collection of tumor samples for biomarker and translational studies [13]. Immunotherapy appears to be especially beneficial in the neoadjuvant setting, as it can potentially result in an effective presentation of multiple neoantigens and better survival outcomes than targeted therapy [9,12,14]. A pooled analysis of six clinical trials (4 ICIs trials and 2 TT trials) provides insight into neoadjuvant treatment and indicates better relapse-free survival (RFS) outcomes in patients treated with ICIs than in patients treated with TT [11,14,15,16,17,18,19]. Furthermore, in that analysis, combined anti-CTLA-4 (ipilimumab) plus anti-PD-1 (nivolumab) immunotherapy had superior efficacy than anit-PD-1 (nivolumab, pembrolizumab) monotherapy [14].

The Cochrane meta-analysis of neoadjuvant clinical trials in melanoma patients did not support the neoadjuvant approach, but since it was carried out, new evidence has emerged in favor of the superior efficacy of the neoadjuvant over the adjuvant approach [20]. The phase II randomized trial, SWOG 1801, compared the adjuvant and neoadjuvant immunotherapy strategies head to head. The latter was found to be superior in terms of event-free survival (EFS): the 2-year EFS was 72% in patients who received neoadjuvant plus adjuvant treatment, while it was 49% in patients treated with adjuvant-only therapy [10]. Both schedules consisted of 18 cycles of pembrolizumab; however, in the neoadjuvant–adjuvant setting, the first 3 cycles were administered before surgery, which was followed by another 15 cycles. In contrast, the adjuvant-only cohort received all 18 cycles of pembrolizumab postoperatively [10]. A phase 3 clinical trial designed to compare the efficacy of neoadjuvant nivolumab (anit-PD-1) plus ipilimumab (anti-CTLA-4) (and in the case of a pathological response less than 90%: subsequent adjuvant nivolumab or dabrafenib plus trametinib, depending on *BRAF* mutation status) with adjuvant-only nivolumab—the NADINA trial (NCT04949113)—is ongoing [21]. The results are expected in 2024. The main aspects of neoadjuvant treatment in advanced melanoma patients have been summarized in Table 1.

The greatest potential risk of the neoadjuvant strategy is that in the case of non-responders, progression of the disease during neoadjuvant treatment can prevent planned curative-intent surgical treatment of localized disease, if the disease becomes unresectable or spreads to distant sites [13]. Therefore, if neoadjuvant treatment is considered, it is especially important to anticipate the response of patients to the treatment and expected disease-free survival and provide them with personalized and optimized therapy—this can be achieved using predictive and prognostic biomarkers [13,22]. 

**Table 1:** Comparison of the systemic neoadjuvant treatment approaches in advanced melanoma patients: BRAF/MEK-targeted therapy and immunotherapy. pCR—pathological Complete Response, RFS—Relapse Free Survival, and OS—Overall Survival.

	Neoadjuvant BRAF/MEK-Targeted Therapy	Neoadjuvant Immunotherapy
Pathological response	In a pooled analysis, the rate of pCR was 47% for targeted therapy and 37% for immunotherapy (not a significant difference). Higher pCR rate has been observed with combination immunotherapy (44%) than with monotherapy (21%) (*p* = 0.023) [14].	
Progression on neoadjuvant treatment (before the surgery)	Has not been reported in upfront resectable patients undergoing neoadjuvant treatment [14,23,24], but occurred in 2 of 21 patients with unresectable disease, treated with neoadjuvant cytoreductive treatment to attain resectability [25].	Has been reported in seven (5%) patients in a pooled analysis (three were treated with combination and four with monotherapy [14]. Another clinical trial reported that 12 (of 154 randomized patients) did not undergo surgery due to progression of the disease [10].
Survival outcomes	Inferior RFS outcomes compared to immunotherapy (at 2 years: 47% vs. 75%). No significant difference in OS compared to immunotherapy [14].	Superior RFS outcomes than with targeted therapy. Longer RFS with combination than monotherapy (80% vs. 59% at 2 years) and OS (96% vs. 76% at 2 years) [14].
Pattern of recurrence	In approximately 60% of patients developing recurrence (regardless of treatment modality), the recurrence was at a distant site (including patients also with simultaneous local recurrence). Patients treated with targeted therapy were more likely to develop intracranial recurrence [14].	
Possible role as preoperative, cytoreductive treatment of prior unresectable melanoma to allow surgical resection.	One phase 2 trial reported that after 8 weeks of targeted therapy in 18 of 21 patients (86%) with prior unresectable disease, the surgical resection was possible. R0 margins were achieved in 17 of 18 cases [25].	No clinical trials investigated the usage of immunotherapy in preoperative, cytoreductive setting.

In general, predictive biomarkers are supposed to predict whether the patient will respond to the specific treatment considered, while prognostic biomarkers allow for the anticipation of survival or the time to disease recurrence [26]. It is expected that when selecting a treatment modality with the highest chance of response in the case of each individual patient—determined by appropriate biomarkers—a higher efficacy of the treatment will be observed [22]. Other potential roles of biomarkers, measured during the course of neoadjuvant treatment, are to support the decision, whether the administration of adjuvant therapy is appropriate or to support the selection of the most effective subsequent therapy, in the case of a recurrent disease [22]. Furthermore, prognostic biomarkers are expected to determine the risk of recurrence of the disease and therefore allow the optimization of the follow-up schedule [9]. They are also useful in patient stratification when interpreting data and designing clinical trials [12].

In a rapidly evolving landscape of neoadjuvant therapy in advanced resectable melanoma, biomarker research is one of the key subjects. The aim of this review is to summarize the most recent advances in the field of prognostic and predictive biomarkers in the neoadjuvant setting for both BRAF/MEK-targeted therapy and immunotherapy. We also highlight the most urgent and promising research directions related to this field.

## 2. Clinical and Pathological Biomarkers

### 2.1. Immune Checkpoint Inhibitors

#### 2.1.1. Pathological Response

**Figure 1:**
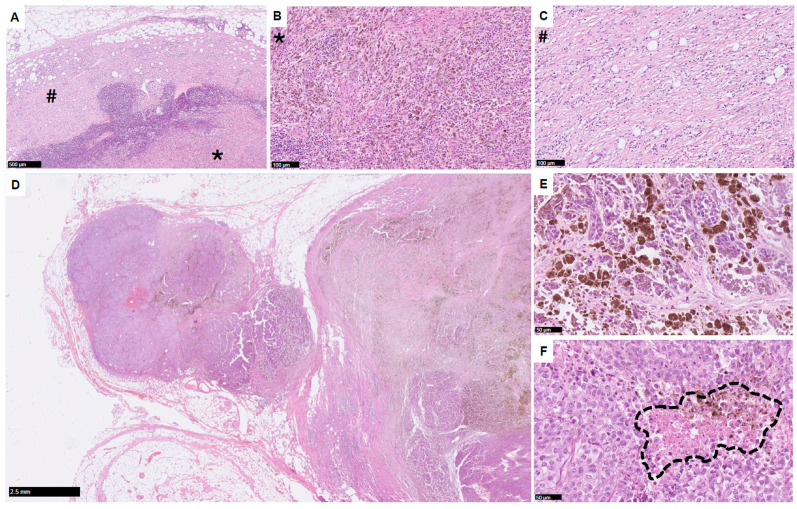
Pathological response to immunotherapy; (**A**) lymph node with both abundant fields of melanophages (40×) with fibrosis (*) and lymphohistiocytic infiltration (#), (**B**) fibrosis in higher magnification (100×) and (**C**) lymphohistiocytic infiltration in higher magnification (100×); (**D**) the typical response “mixture” of different reactions (20×), (**E**) sometimes the melanophages present very dense melanin (400×), which could be diagnostically challenging; (**F**) necrosis can be massive or only focal–marked with dashed line (400×).

Currently, a key reliable biomarker of response to the neoadjuvant ICI treatment is the status of pathological response, assessed in the surgical specimen (Figure 1), as it was shown in a pooled analysis of four clinical trials of neoadjuvant ICIs [14]. It was found that patients who achieved at least a 50% pathological response had improved RFS compared to those who had less than a 50% pathological response: 2-year RFS rates were 96% vs. 37%, respectively [14]. Importantly, a similar RFS was observed in patients with 10–50% of the residual viable tumor compared to those with a near-complete (<10% residual viable tumor) or complete pathological response [14]. In patients treated with neoadjuvant PD-1 and LAG-3 blockage (nivolumab plus relatlimab), the obtained pathological response was also associated with improved RFS [27]. Given this, pathological response has emerged as a surrogate endpoint for RFS [14].

#### 2.1.2. Radiological Response

Radiological response, according to RECIST (Response Evaluation Criteria in Solid Tumors), has also shown prognostic value, as patients who achieved Complete Response (CR) or Partial Response (PR) had an excellent prognosis given the 2-year RFS rates of 100% and 96%, respectively [14]. In one study, although the radiological response generally correlated with the pathological response, 38% of patients with Stable Disease (SD) achieved a complete pathological response [14]. In another phase 2 trial with neoadjuvant nivolumab and relatlimab, the pathological and radiological responses were often disconcordant [27]. In these studies, a trend toward underestimation of the response by a radiological assessment can be seen. However, a radiological response potentially enables additional risk stratification in patients with less than a 50% pathological response [14].

#### 2.1.3. Baseline Clinical/Demographic Characteristics

Baseline characteristics, including sex, geographic region, AJCC stage, *BRAF* mutational status, time to surgery, and site and extent of nodal metastases, were not associated with the attainment of a complete pathological response, although on multivariable analysis, patients with stage IIIC disease had worse RFS outcomes than those with stage IIIB, while a younger age was associated with a poorer prognosis for RFS [14]. Of note, in the adjuvant setting, the AJCC-7/8 stage was found to have prognostic, but not predictive, value [28,29]. The AJCC-8 stage was used (together with the lactate dehydrogenase (LDH) level) as a stratifying variable in the SWOG 1801 neoadjuvant trial [10] and still represents a key risk assessment in melanoma patients.

#### 2.1.4. Other Factors

Although the occurrence of immune-related adverse events has been linked with improved survival in patients with an advanced metastatic disease [30], in the neoadjuvant setting, the maximum grade (G3/4) of immune-related adverse events was not associated with a pathological response rate in the OpACIN-neo clinical trial [17]. However, an association of emotional distress at baseline with a decreased rate of pathological response and poorer RFS outcomes was described in the neoadjuvant setting [30]. Another factor which predicted the pathological response to neoadjuvant nivolumab plus ipilimumab, was a gut microbiota profile [31]. Concordant findings on gut microbiota association with response to the treatment were reported in patients with advanced metastatic melanoma treated with palliative anti-PD-1 immunotherapy, suggesting that gut microbiota may become an additional predictive biomarker for response to the immunotherapy [32].

### 2.2. BRAF/MEK Inhibitors

#### 2.2.1. Pathological Response

Two phase 2 clinical trials investigated neoadjuvant-targeted therapy with BRAF/MEK inhibitors (dabrafenib and trametinib) [15,16]. In a pooled analysis of these trials (and additional 10 more patients), 48% of patients achieved a pathological complete response (pCR) and had a 63% 2-year RFS, while those with a pathological noncomplete response were only 24% [33]. Another pooled analysis of neoadjuvant ICIs and targeted therapy trials showed that, unlike patients treated with neoadjuvant ICIs (in whom the achievement of any pathological response >50% improves RFS outcomes), in those who receive targeted therapy, only the pCR is a reliable prognostic factor for improved RFS [14]. In patients with a residual viable tumor of 10–50% after neoadjuvant-targeted therapy, the prognosis of RFS is rather similar to those with a >50% residual viable tumor and, thus, significantly worse than in the case of a complete pathological response [14,33]. In our practice in individuals experiencing a major pathological response, characterized by the absence or presence of less than 10% viable cells within the tumor, both median disease-free survival and progression-free survival were significantly extended compared to those observed in individuals with a minor pathological response [24]. Additionally, the histopathological characteristics of the tumor bed/residual tumor after neoadjuvant therapy may yield a prognostic value. In patients with a complete pathological response and hyalinized/mature fibrosis, the 2-year RFS was 76%, while in those with a complete pathological response and no hyalinized/mature fibrosis, the 2-year RFS was 29%. Hence, this factor could potentially stratify patients with pCR to neoadjuvant-targeted therapy into low- and high-risk groups and possibly guide further clinical decisions (e.g., whether the adjuvant part of the therapy is necessary to maintain the benefit).

#### 2.2.2. Radiological Response

It is not clear whether the complete pathological response can be accurately predicted by radiological response during the course of neoadjuvant TT treatment. In the NeoCombi phase 2 trial, the complete pathological response was consistent with both the complete radiological response according to RECIST (CR) and the complete metabolic response (assessed by FDG-PET) [16]. In contrast, in other studies, the correlation between radiological response according to RECIST and pathological response was not found [23,25].

#### 2.2.3. Baseline Clinical Characteristics

In patients treated with neoadjuvant BRAF/MEK inhibitors, none of the baseline characteristics (including sex, geographic region, stage of AJCC, time to surgery and site, and extent of nodal metastases) were associated with the attainment of pCR [14]. The baseline factors (including the clinical stage) also did not influence RFS [33]. However, the significance of these observations is limited by relatively small sample sizes among clinical trials of neoadjuvant BRAF/MEK inhibitors. Importantly, in an advanced metastatic setting, several baseline clinical biomarkers are prognostic factors for PFS and OS, including the number of metastatic foci, the performance status of the Eastern Cooperative Oncology Group (ECOG), sex, age, and LDH activity [34,35]. Although some of them are rather specific for an advanced metastatic setting, others, e.g., LDH activity, may also be useful in the context of neoadjuvant therapy, but this needs to be confirmed. A summary of the most promising clinical and pathological biomarkers (in both the immunotherapy and targeted therapy contexts) can be found in Table 2.

## 3. Molecular and Immune Biomarkers

### 3.1. Immune Checkpoint Inhibitors

#### 3.1.1. IFN-γ Signature

**Figure 2:**
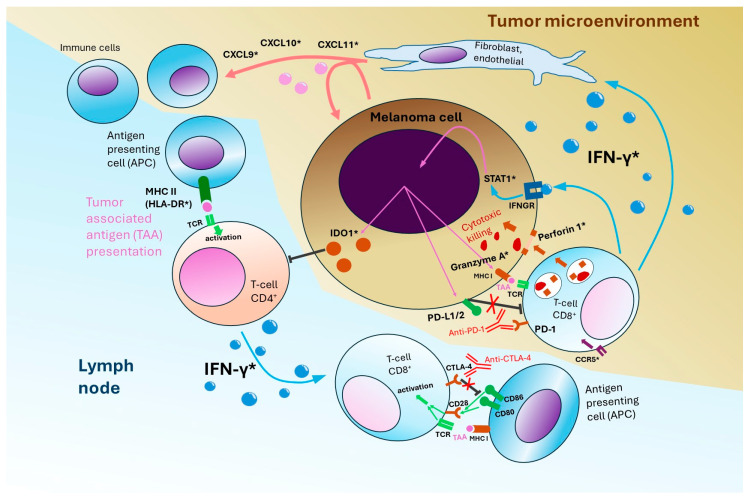
A role of interferon-gamma signaling in the development of anti-tumor response during anti-PD-1 plus anti-CLTA-4 immunotherapy. The impact of chemokines CXCL9, CXCL10 and CXCL11 on antigen-presenting cells (APC) depicted. Tumor-associated antigen (TAA) presentation on MHC II (HLA-DR) molecule to CD4^+^ T-cells. Molecules marked with (*) include translation products of mRNAs, which are measured to determine a 10-gene interferon-gamma (IFN-γ) signature score, including Perforin 1 and Granzyme A involved in cytotoxic melanoma cell killing by CD8^+^ T cells.

Although no molecular biomarker is currently validated to predict immunotherapy responses in patients with melanoma, certain biomarkers are emerging as potentially reliable predictive or prognostic factors. A biomarker analysis of phase 1b OpACIN and phase 2 OpACIN-neo clinical trials (of neoadjuvant nivolumab plus ipilimumab) revealed promising predictive values of the IFN-γ signature score and tumor mutational burden (TMB) in the baseline tumor sample [36]. The IFN-γ signature score is defined as a measure of the expression of ten genes related to IFN-γ (Figure 2) and has previously been shown to be able to predict response to anti-PD-1 treatment in patients with advanced melanoma [37]. Additionally, a high level of the IFN-γ signature indicates a favorable prognosis in patients treated with adjuvant immunotherapy [29]. 

The IFN-γ score has a predictive value as an individual biomarker [36,38] or when combined with other molecular biomarkers, which will be discussed later. Patients with a low IFN-γ score are less likely to achieve a pathologic response and are more prone to disease recurrence, compared to those with a high IFN-γ score [36,39]. Based on these findings, the phase 1b DONIMI trial was conducted, in which the selection of the treatment regimen was determined by the baseline IFN-γ signature score [40]. Patients with IFN-γ-low tumors (who were less likely to benefit from single immunotherapy) were randomized to regimens consisting of domatinostat and nivolumab with or without ipilimumab. Simultaneously, patients with IFN-γ-high tumors (more likely to respond to ICI treatment) were randomized to nivolumab with or without domatinostat. Thus, in potential responders, treatment decreased, while in potential non-responders, it intensified [40]. Domatinostat is a class I histone deacetylase 1 inhibitor, and its effect on inducing changes in the tumor immune microenvironment, supporting a response to ICIs, has been reported [41]. The design of the DONIMI clinical trial is an example of a personalized biomarker-driven treatment strategy. The advantages of this approach are maximizing the rate of responders (through the intensification of the treatment in patients with a refractory disease), while simultaneously minimizing the incidence of serious adverse events (through non-exposure to the aggressive and highly toxic regimens, those who will likely respond to the de-escalated therapy). Although the results of the DONIMI trial did not show a benefit in adding domatinostat to the ICI on the pathological response rate, the most important finding of this study is that in patients with IFN-γ-high tumors, neoadjuvant systemic therapy can decrease, while maintaining a pathological response rate [41]. This particular clinical trial did not randomly select patients for nivolumab plus ipilimumab in any of its arms, which should be considered a limitation. However, a cross-trial comparison demonstrates that in patients with IFN-γ-high tumors, after neoadjuvant anti-PD-1 monotherapy or a combination with anti-CTLA-4 therapy, the pathological response rates are similar [39,41]. The longitudinal biopsy sample analysis during neoadjuvant treatment in the DOMINI clinical trial has also shown that the tumor transition from IFN-γ-low to IFN-γ-high, at the third week of treatment, allows for the achievement of a pathological response in 50% of the patients treated with domatinostat plus nivolumab and 80% of the patients treated with domatinostat with nivolumab plus ipilimumab [40]. Instead, all patients with tumors that were IFN-γ-low at both the baseline and the third week of treatment were not responders at the time of surgical resection at week six [40].

In addition to the 10-gene IFN-γ signature score, other signatures of inflammatory gene expression have been proposed as predictors of a response, mostly in an advanced metastatic setting. For example, a four-gene signature (including CD274, CD8A, LAG3, and STAT1) was shown to predict the outcome of immunotherapy-treated patients in an advanced metastatic setting [42].

#### 3.1.2. Tumor Mutational Burden

The tumor mutational burden—expressed as mutations per megabase (mut/Mb)—is the total number of mutations per coding area of a tumor genome [43]. It correlates with the objective response rate (ORR) in patients treated with anti-PD-1/anti-PD-L1 agents for different tumor types, including metastatic melanoma [43]. TMB has been reported as a potential predictive biomarker in patients with advanced melanoma, treated with ICI, in whom a high level of TMB was associated with a superior efficacy of ICI treatment [42,43]. A high level of TMB was also a positive prognostic factor in patients treated with adjuvant immunotherapy [29]. Existing evidence indicates that TMB (especially when combined with other biomarkers) may predict the response to neoadjuvant immunotherapy. In the neoadjuvant setting, all patients treated with anti-PD-1 plus anti-CTLA-4 combination immunotherapy, who had a high TMB and IFN-γ score, achieved a pathologic response [36]. In contrast, patients with a low TMB and a low IFN-γ score had a response rate of 39% [36]. Patients with a low IFN-γ score and a high TMB had a pathological response in 89% of cases, while patients with a high IFN-γ score and a low TMB had a response rate in 91% of cases [36]. Similar observations have been reported confirming the predictive value of the combined assessment of the TMB assessment and IFN-γ that have been reported in other neoadjuvant immunotherapy trials [44]. However, despite its potential value as a predictive biomarker, TMB has several limitations, most significant of which are the inconsistency of the cut-off values and the multiplicity of assays used for the TMB measurement [45,46]. Furthermore, in the neoadjuvant setting, the evaluation of TMB (TMB is preferably calculated by whole-exome sequencing—WES) appears to be not fast enough to be a widely acceptable predictive biomarker, used for the selection of first-line treatment, which should not be delayed by a long turnaround time of WES [46,47]. It should be noted that—in order to make the TMB valid and available for routine clinical practice—efforts must be made and are ongoing to harmonize and optimize the TMB evaluation [46].

#### 3.1.3. Expression of PD-L1 and LAG-3

In the DONIMI clinical trial, the PD-L1 score (PD-L1 Tumor Proportion Score) predicted the response to treatment: All patients with a baseline PD-L1 score > 50% had a pathological response, while of those with a 1–50% PD-L1 score, 58% had a pathological response, and in those with a baseline PD-L1 score < 1%, only 22% had a pathological response [40]. In addition, a PD-L1 score less than 50% at the third week of treatment predicted the lack of a pathological response [27,40]. Similarly, in the PRADO trial of neoadjuvant nivolumab plus ipilimumab, the baseline expression of PD-L1 was correlated with a pathological response rate; that is, for patients with >50% PD-L1-expressing tumor cells, the pathological response rate was 100%; for those with a 1–50% PD-L1-expressing tumor cells, the pathological response rate was 92%, and for those with less than 1% PD-L1-expressing tumor cells, the pathological response rate was 56% [48]. On the contrary, in the OpACIN neo trial, PD-L1 expression was not correlated with a rate of pathological response to anti-CTLA-4 plus anti-PD-1 treatment [17]. In the adjuvant setting, PD-L1 expression below 5% was associated with poorer survival outcomes [29]. These findings indicate that despite having some predictive value, the expression of PD-L1 does not fully differentiate responders and non-responders to immunotherapy, and therefore should be interpreted in the context of other biomarkers, rather than as an individual factor. PD-1 and LAG-3 levels in baseline tumor samples were not associated with a pathological response rate to anti-LAG-3 (relatlimab) plus anti-PD-1 treatment (nivolumab) [27]. It is noteworthy that in the clinical trial of nivolumab plus relatlimab (compared to nivolumab monotherapy) in an advanced metastatic setting, an LAG-3 expression below 1% was associated with shorter PFS, but treatment was beneficial regardless of the LAG-3 expression [49].

#### 3.1.4. Tumor Microenvironment

The phase 2 trial of neoadjuvant nivolumab plus ipilimumab revealed that higher CD8-positive T cell infiltrates in baseline tumor samples were associated with the pathological response to treatment [19]. That analysis also showed a significant association of the pathological response with the expression of Granzyme B, FoxP3, and PD-1 at baseline [19]. In the phase 2 clinical trial of neoadjuvant nivolumab plus relatlimab, an association was found between a higher frequency of CD45-positive cells in the tumor at baseline and the pathological response [27], while in the phase 2/3 trial of this combined immunotherapy in an advanced metastatic setting, the response to treatment was associated with a higher number of CD8-positive T cells positive for PD-1 and positive for ICOS [50]. The immune cell signature, including the B-cell signature, was found to predict a response in neoadjuvant ICIs in a small study [51].

#### 3.1.5. Circulating Biomarkers

In terms of the circulating biomarkers studied in the neoadjuvant setting, in patients treated with neoadjuvant nivolumab plus ipilimumab, higher baseline levels of vascular endothelial growth factor receptor 2 (VEGFR-2), fractalkine, also known as chemokine ligand 1 (CX3-C motif) and programmed cell death 1 ligand 2 (PD-L2), were associated with a lower rate of pathological response [36]. The expression of VEGFR-2 may be a biomarker of particular interest, as axitinib (inhibitor of VEGFR 1-3, c-KIT, and PDGFR) has shown activity in patients with melanoma either as a single agent [52] or in combination with toripalimab (anti-PD-1)—the latter regimen was investigated in patients with mucosal melanoma [53]. In the adjuvant setting, the higher baseline C-reactive protein (CRP) serum level was associated with a decreased RFS [29], while in patients treated with neoadjuvant immunotherapy, a higher baseline CRP level was related to a gut microbiota profile, which was associated with a poorer survival outcome [31]. 

Currently, new circulating molecular biomarkers are emerging as potentially applicable in clinical use, including the circulating tumor cell (CTC) and circulating tumor DNA (ctDNA). These biomarkers were shown to correlate with the tumor burden in advanced stages of melanoma, and they also provide prognostic information [54,55,56]. However, they still must face several limitations, including a lack of cut-off values, despite the fact that sole detectability (compared to undetectability) of these biomarkers already seems to be a prognostic factor [54,55,56]. The circulating biomarkers appear to be particularly important in pre-treatment predictive and prognostic assessments, as liquid biopsy becomes a common and reliable diagnostic tool [55] and, in future, might become an alternative for invasive, tissue-based biomarkers. Liquid biopsy also seems more useful for longitudinal monitoring of the treatment response (for example, Long et al. monitored ctDNA longitudinally during the neoadjuvant-targeted therapy, but found no significant correlations with baseline characteristics or outcomes, presumably due to the small sample size [16]). Of note, a potential limiting factor for the application of a liquid biopsy in neoadjuvant settings could be the undetectability of circulating molecular biomarkers in patients with a low tumor burden, considered for curative-intent preoperative treatment and surgery. Further research in the field of liquid biopsy in melanoma patients is warranted, especially in the neoadjuvant context. 

#### 3.1.6. Complex Biomarker Assays

As new predictive biomarkers are under development and are undergoing validation (Table 3), another possibility to obtain predictive systems is combining existing reliable biomarkers into composite, multi-biomarker scores. The combined IFN-γ score and the TMB assessment, which showed an impressive prognostic value in patients treated with neoadjuvant immunotherapy, has been described in detail above, in this chapter [36]. Interestingly, a composite score of three biomarkers has also been proposed, including the expression of PD-L1 in addition to the TMB and IFN-γ score [57]. In patients with high levels of all three biomarkers, 100% three-year event-free survival (EFS) and OS were reported, while for those with low levels of all three biomarkers, the three-year EFS and OS rated were 56% and 72%, respectively [57]. Furthermore, based on findings made in advanced metastatic settings, the introduction of the composite biomarker assay has been suggested, proposing PD-L1, TMB, and inflammatory gene expression signatures as components [42]. Further exploration of possible composite biomarker scores can lead to the development of robust predictive (and prognostic) assays, useful in routine practice and also in clinical trial design.

### 3.2. BRAF/MEK Inhibitors

#### 3.2.1. *BRAF* Mutational Status

The leading molecular biomarker in the context of a targeted therapy in patients with melanoma is the presence of the *BRAF*V600 mutation, as the role of BRAF/MEK inhibitors is well established in adjuvant or palliative therapy in patients with *BRAF*-mutated tumors [7,8,58]. Although the correlation of the *BRAF*V600E or *BRAF*V600K variants with the pathological response was not addressed in neoadjuvant trials, in an advanced metastatic setting, the *BRAF*V600E-only tumor genotype was associated with favorable PFS and OS compared to the V600K-only genotypes or V600K and V600E in a multivariate analysis [34,35]. Although the most common variants are BRAFV600E/K, the responsiveness to the inhibition of BRAF/MEK in patients with variants other than *BRAF*V600E/K, such as rare *BRAF* mutations, has also been reported in advanced metastatic setting [59]. However, in clinical trials of targeted neoadjuvant therapy (and preoperative cytoreductive)-targeted therapy, only patients with *BRAF*V600E/K mutations were enrolled [15,16,25] and thus the issue of rare *BRAF* mutations in the neoadjuvant setting and their correlation with a pathological response still needs to be addressed.

#### 3.2.2. TMB and IFN-γ Expression Signature

In a phase 3 clinical trial of adjuvant dabrafenib and trametinib (BRAF and MEK inhibitors, respectively) in patients with resected stage III melanoma, the prognostic value of the five-gene IFN-γ expression signature and the TMB was investigated [60]. The evidence indicated that a high TMB was associated with a favorable prognosis in the placebo group, but not in the dabrafenib plus trametinib group, while a high five-gene IFN-γ signature was prognostic for prolonged RFS in both groups [60]. But another important finding was that patients with a high TMB and a low expression of the five-gene IFN-γ signature obtained the least benefit from adjuvant therapy compared to those with a low expression of the TMB and a high expression of the five-gene IFN-γ signature, who derived the greatest benefit from adjuvant treatment [60]. It still needs to be validated whether the combined biomarkers, TMB and five-gene IFN-γ signature, can identify patients who will benefit from adjuvant therapy. However, these findings indicate the signature of the TMB and the five-gene IFN-γ signature as potential predictive biomarkers in the neoadjuvant setting, but warrant further research.

#### 3.2.3. Other Biomarkers

In terms of the other molecular biomarkers, in one phase 2 clinical trial, patients who had a lower pERK expression in baseline tumor samples had a higher complete pathological response rate than those with a higher pERK expression [15], and in another phase 2 trial with a larger cohort undergoing targeted neoadjuvant therapy, such an association was not observed [16], and the same applied for a retrospective study in eight patients [23]. Confirmation of pERK involvement (in resistance to BRAF/MEK inhibition in the neoadjuvant setting) could result in the introduction of ERK inhibitors to the systemic treatment of patients with overexpressed pERK and could possibly lead to the overcoming of resistance. Additionally, the second of the clinical trials mentioned above showed a higher rate of complete pathological responses among patients with a higher proportion of Ki-67-positive or PD-L1- and SOX10-positive melanoma cells in the baseline tumor samples [16]. In this trial, the increase in tumor-infiltrating CD8-positive T cell density was also associated with the attainment of a complete pathological response [16]. In contrast, another study did not show differences in intratumoral CD8-positive T cell density between complete pathological responders and noncomplete responders, but an increase in *TIM-3* and *LAG-3* expression in CD8-positive PD-1-positive T cells was correlated with the noncomplete pathological response [15]. The detectability of circulating tumor DNA (ctDNA) at baseline was not correlated with the pathological response or recurrence-free survival [16].

## 4. Conclusions and Further Directions

Neoadjuvant immunotherapy is emerging as a highly effective treatment approach and may become the new standard of care in patients with resectable stage III/IV melanoma. Contemporary data suggest that it has superior ability to prevent the recurrence of a melanoma than adjuvant treatment—the current standard of care. Neoadjuvant-targeted therapy, which could be available only for patients with *BRAF*-mutated tumors, appears to produce worse long-term survival outcomes than neoadjuvant immunotherapy, but may be beneficial in patients with unresectable disease as a cytoreductive treatment, making the patient feasible for surgery after shrinkage of the lesions. Current evidence does not support the use of any biomarker as a robust predictive factor for the response to immunotherapy. Out of the molecular biomarkers, tumor mutational burden, 10-gene IFN-γ signature, and PD-L1 expression are emerging as potential predictive biomarkers for responses to immunotherapy. An aggregation of these individual biomarkers into composite assays may yield a better predictive value. The pathological response is the most reliable prognostic factor in patients who underwent a resection after neoadjuvant treatment. In patients treated with immunotherapy, any pathological response greater than 50% seems to improve relapse-free survival, while in patients treated with targeted therapy, only the achievement of a complete pathological response seems to improve survival outcome. 

As the safety and efficacy of the neoadjuvant approach are currently being investigated, predictive and prognostic biomarkers warrant further research, including prospective validation in large-scale studies. The most urgent directions of research in this field are prospective validation of the proposed and emerging biomarkers, creation of complex biomarker assays (which would maximize the potential of already proposed biomarkers by combining them into composite scores), analysis of treatment resistance mechanisms (brought by a unique ability of the neoadjuvant setting to obtain post-treatment specimen), exploration of new fields specific to the neoadjuvant setting (e.g., recurrence risk stratification beyond pathological response or surgical treatment de-escalation in the responders), and application of novel technologies (e.g., liquid biopsy) in routine clinical practice. Novel biomarkers, such as micro-RNAs (miRNAs) or extracellular vesicles (EVs), which are being investigated due to their potential diagnostic/follow-up monitoring application [61,62], can also make their way to the utilization as prognostic/predictive biomarkers in neoadjuvant therapy. For example, EV profiling in exudative seroma after surgical resection can serve as biomarker of the melanoma progression [63], but these findings need to be confirmed in patients undergoing resection after neoadjuvant treatment. Interesting observations have been made in patients with non-small cell lung cancer, whose response to neoadjuvant immunotherapy could be predicted by plasma EV long RNAs, once again highlighting the potential of liquid biopsy [64]. In the future, the biomarker-driven personalization of treatment for patients with resectable stage III/IV melanoma is expected to produce maximum efficacy at the lowest possible risk of serious adverse events and limited surgical morbidity. 

## Figures and Tables

**Table 2:** Summary of advantages and limitations of clinical and pathological biomarkers in neoadjuvant immunotherapy and targeted therapy.

	Advantages	Limitations
Pathological response	Is a reliable prognostic biomarker for RFS. Has been proposed as surrogate endpoint for RFS in clinical trials. In immunotherapy patients, any level of pathological response >50% is related with favorable prognosis, whereas in targeted therapy patients, only the complete pathological response is a prognostic factor for increased RFS [14]. Histopathological features of the response to targeted therapy provide additional risk stratification [33].	Can be obtained only after surgery. Further search for additional risk stratification for all levels of pathological response is warranted.
Radiological response	May provide additional risk stratification beyond pathological response [14].	Both in immunotherapy and targeted therapy studies a discordance with pathological response has been observed [23,25,27].
Gut microbiota profile	Correlation between gut microbiota profile and pathological response to immunotherapy has been observed [31]. Does not require invasive procedures to be assessed. Can be modified by dietary intervention/fecal transplant.	Although gut microbiota profile influences the response to immunotherapy, it is believed that tumor-intrinsic factors are main determinants of the response to the treatment [31].

**Table 3:** Advantages and limitations of the emerging predictive biomarkers of response to neoadjuvant immune checkpoint inhibitors (ICIs).

Biomarker	Advantages	Limitations
IFN-γ signature	Baseline IFN-γ signature expression score correlates with response to ICIs [37,39]. IFN-γ signature score-driven treatment personalization yielded promising results [40]. Possibly, this biomarker can become a reliable factor for neoadjuvant treatment personalization. Possible component of a composite biomarker assay.	Obtaining high-quality tumor biopsy sample to perform RNA sequencing is mandatory.
Tumor mutational burden (TMB)	Promising results support the role of TMB as a predictive biomarker, especially when combined with other biomarkers [36]. Possible component of a composite biomarker assay.	Inconsistency of cut-off values [46]. Time-consuming method. High-quality tumor biopsy samples are required.
PD-L1 expression	High expression in baseline tumor samples of PD-L1 correlates with pathological response [40,48]. Possible component of a composite biomarker assay.	Lack of correlation between PD-L1 expression and pathological response to combination immunotherapy has also been reported, patients with low PD-L1 expression also benefit from ICI treatment [36].

## Data Availability

Not applicable.
